# The Efficacy of Conservative Management in Uncomplicated Acute Appendicitis - A Single-Center Retrospective Study

**DOI:** 10.7759/cureus.32606

**Published:** 2022-12-16

**Authors:** Hira F Akbar, Talha Kareem, Nitasha Saleem, Muhammad I Seerat, M. Irshad Hussain, Irfan Javed, Syed Muhammad Ali

**Affiliations:** 1 General Surgery, Recep Tayyip Erdogan Hospital, Muzaffargarh, PAK; 2 General Surgery, Hamad General Hospital, Doha, QAT; 3 General Surgery, Recep Tayyip Erdogan Hospital, Muzzafargarh, PAK; 4 Surgery, Weill-Cornell Medical School, Doha, QAT; 5 Acute Care Surgery, Hamad General Hospital, Doha, QAT

**Keywords:** covid 19, emergency appendectomy, a retrospective study, conservative management, uncomplicated apendicitis

## Abstract

Background

Acute appendicitis remains the most common cause of lower abdominal pain leading to emergency visits. Even though the standard treatment of acute appendicitis remains appendectomy, in recent times, multiple randomized control trials and meta-analyses have deduced conservative treatment as a successful alternative treatment. During the coronavirus disease (COVID) pandemic, with a shortage of staff and resources, treatment with conservative management of uncomplicated acute appendicitis became very beneficial under certain circumstances and conditions. This study aimed to assess whether it is effective to manage patients with uncomplicated acute appendicitis with antibiotic therapy.

Methodology

This was a single hospital based retrospective, cross-sectional study from Jan 2015 to May 2020. Patients with clinical and radiological features of uncomplicated acute appendicitis with Alvarado's score >6 were included in the study. Patients were kept on antibiotics, intravenous fluids, and analgesia as part of a conservative regime. Those who failed to respond to conservative therapy were managed surgically. The follow-up period was six months.

Results

One hundred eighty-two cases of uncomplicated acute appendicitis were included and managed conservatively, of which 52.2% were males while 47.8% were females. The median age of the patients was 26 years. Conservative treatment was successful in 26.2% of the patients, with a recurrence of 5.5% in the six-month follow-up period. The mean number of days of hospital stay was three days in patients treated with conservative or surgical treatment.

Conclusion

Conservative management is gaining popularity, and many centers are inclined towards non-operative management; however, appendectomy remains the gold standard treatment for appendicitis.

## Introduction

Acute appendicitis has been the most common cause of lower abdominal pain leading to emergency visits worldwide [1]. To avoid major complications, appendectomy has remained the standard treatment for uncomplicated acute appendicitis for over a century [2]. For decades, a significant debate has been going on regarding the management of uncomplicated acute appendicitis. Multiple meta-analyses and clinical trials have been published to find the best approach for managing uncomplicated acute appendicitis, but no such consensus has been reached [3-5].

In 2016, a meta-analysis of randomized control trials (RCT) concluded appendectomy was the most effective treatment, with more efficacy and low complication rates. Still, it also stated that a subgroup of uncomplicated acute appendicitis could benefit from antibiotic therapy [6]. Similarly, in 2018, APPendicitis ACuta III (APPAC III) conducted an RCT which showed that antibiotic therapy alone could be an alternative to appendectomy when patients treated early with antibiotics showed recurrence of 39% in five years of follow-up [7].

RCT conducted by Salminen et al. showed that 72.7% of the patients did not require surgery even at follow-up, making the treatment efficacy between both groups about 27%. With the prescribed noninferiority margin of 24%, the study was unable to demonstrate the noninferiority of antibiotic treatment to surgery [8]. Similarly, an RCT in Pakistan showed that both management were comparable, with 90.62% efficacy at one month, decreasing to 71.87% efficacy at one year [9].

In 2020, the whole world was in the midst of a pandemic caused by the virus SARS-CoV-2. Due to the significant inflow of patients with COVID emergencies, ways to defer other non-absolute emergencies were sought. Non-surgical management in uncomplicated acute appendicitis during times like these could be very beneficial when access to operating theatres and the medical personnel needed for delivering these services are limited [10]. Findlay published an online article in 2020 where he discussed and compared the management regimes for acute appendicitis in light of the coronavirus disease (COVID) pandemic. He concluded that a trial of antibiotic therapy could be successful. He also shared the possibility that the COVID pandemic might shift the whole paradigm for treating acute appendicitis [5].

The aim of this study is to assess whether it is effective to manage patients with uncomplicated acute appendicitis with antibiotic therapy. We hypothesize that most patients undergoing conservative management would be successfully treated; however, failure of conservative management would be associated with increased morbidity and prolonged hospital stay.

## Materials and methods

A retrospective review of medical records was carried out in our institute after the approval of the institutional review board. All electronic data of admitted patients from January 2015 to May 2020 for patient demographics, laboratory results, radiological findings, and antibiotic therapy for conservative management of uncomplicated acute appendicitis were searched. Privacy of the patient and data protection was done by keeping the identity of the patients anonymous by excluding the medical registration (MR) number or names of the patient throughout the study. In this retrospective study, data from previous records were used, and patients were already treated in the hospital, so no informed consent from the patient was required.

All patients between the age of 10-65 years, admitted with the diagnosis of uncomplicated acute appendicitis as confirmed on ultrasound (sensitivity 81% and specificity 88%) irrespective of sex and managed conservatively were included in the study [11]. Uncomplicated acute appendicitis was defined as patients with the Alvarado's score of 6 [12] or higher with the ultrasound findings of an inflamed but grossly intact appendix (done by a radiologist with an experience of two years post fellowship) and no associated findings of abscess and peritonitis clinically. All patients who initially underwent surgical intervention at presentation, with complicated appendicitis, any known co-morbidities, immunocompromised, and previous lower abdominal surgeries as recognized through history and previous medical records, were all excluded from our study. Being a resource-limited country, none of the patients underwent a CT scan abdomen for the diagnosis of uncomplicated acute appendicitis, following the hospital policies.

A total of 182 patients were included in the study based on the above-mentioned inclusion and exclusion criteria. All patients were kept nil per mouth (NPO) till the settlement of vomiting. At the same time, they received intravenous (IV) injections of cefuroxime 750mg and metronidazole 500mg three times a day, injection of paracetamol 1g four times a day, and infusion diclofenac 75mg two times a day for at least 24 hours. When patients were kept NPO, they were given injectable fluids, and a six-hourly recording of; temperature, blood pressure, pulse rate, respiratory rate, and the local abdominal sign was done. Patients who improved were discharged the next morning on oral antibiotics (tablets ciprofloxacin 500mg and metronidazole 500mg two times a day) and oral painkillers (tablets paracetamol 1g three times and Brufen 400mg two times daily) for seven days. Patients who clinically deteriorated or did not respond to the conservative treatment were operated on with either open or laparoscopic appendectomy. Patients with repeated disease symptoms, like persistent pain in the right iliac fossa, leukocytosis, and anorexia, and ultrasound findings of an inflamed appendix on a follow-up visit or in an emergency within six months after successfully managing conservatively, were labeled recurrent appendicitis.

The effectiveness of conservative treatment was defined as clinical resolution of all symptoms without the need for surgical intervention, along with tolerating oral diet and no recurrence within six months of follow-up.

Failure of treatment was divided into two sections. Firstly, patients who showed a lack of clinical improvement in their symptoms, i.e., persistent pain in the right iliac fossa, persistently increased leukocyte count, and patients who required surgical intervention. Secondly, patients who were admitted again with repeated symptoms of acute appendicitis within the period of six months of follow-up had undergone an appendectomy.

Frequencies and percentages were calculated for patient characteristics and other categorical variables, while medians for continuous data were calculated. The Chi-square test (or fisher exact test when appropriate) was used to test the significance of association for the discrete variable. At the same time, an independent t-test was applied to find the relation between numerical data. A p-value of <0.05 was considered significant. All statistical analysis was performed using Statistical Package for the Social Sciences version 23.0 (SPSS; IBM Inc., Armonk, New York). All tables and figures were made using Microsoft Excel 2016 (Microsoft, Redmond, Washington).

## Results

In this study, 182 cases of uncomplicated acute appendicitis were included and managed conservatively, of which 95 (52.2%) were males. In contrast, 87 (47.8%) were females showing an almost equal occurrence of uncomplicated acute appendicitis among gender. The median age of the patients was 26 years. No significant difference was noted between the gender and effectiveness of conservative treatment in patients with uncomplicated acute appendicitis. 

Among these 182 patients, conservative treatment was effective in only 48 patients (26.4%), with no treatment failure or recurrence in the next six months of treatment. A significant difference was noted between the effectiveness and recurrence of the symptoms (p<0.05). More than half of the patients had a failure of conservative treatment (n=133, 73.1%). There was a significant difference (p=0.000) between the conservative treatment's effectiveness and the surgical intervention requirement. 

**Figure 1:**
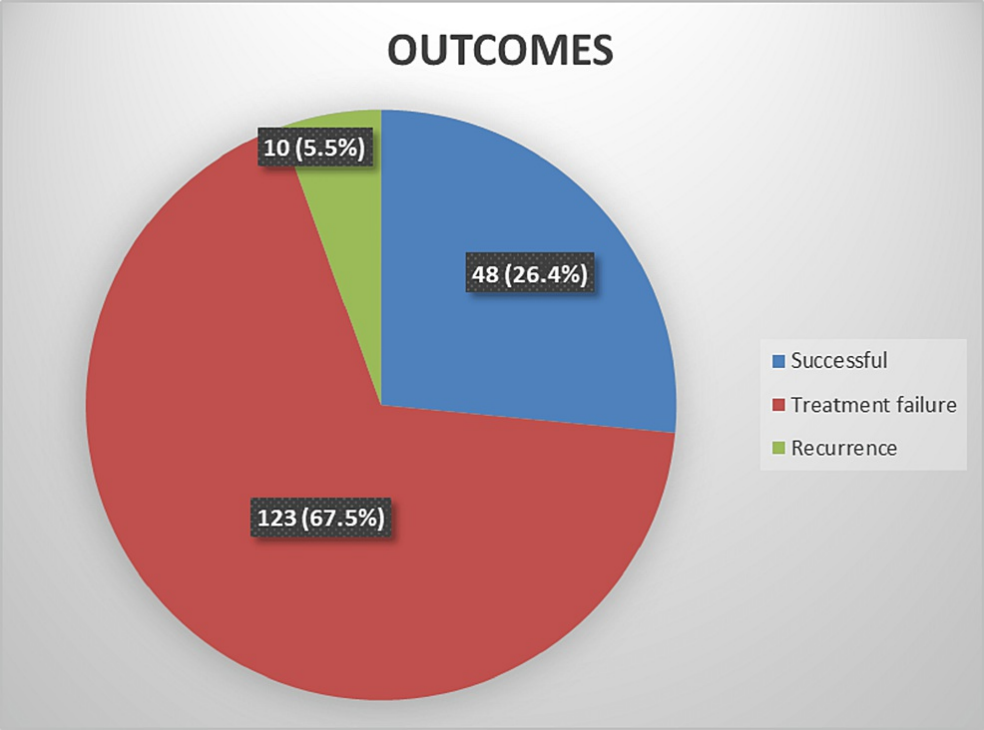
Outcomes of conservative management

One hundred twenty-three patients failed conservative treatment on the initial admission and underwent surgical intervention, while ten patients (5.5%) had treatment failure and developed recurrence during six months of follow-ups (Figure 1). 

The mean hospital stay between patients with effective conservative treatment and those who had failed treatment and underwent surgical intervention was three days. No significant difference was noted between these two groups (p=0.551). 36.7% of patients treated successfully with conservative treatment had a white cell count of less than 12X10^3^. However, no significant difference was observed between white cell count and the effectiveness of conservative treatment.

Table 1 contains the correlation of different variables with the efficacy of the conservative treatment.

**Table 1:** Relation of variables with the efficacy of conservative treatment * statistically significant

Variable	Efficacy of Conservative Treatment		p-value
	Successful	Unsuccessful	
Gender			0.869
Male	25	70	
Female	24	63	
Recurrence of symptoms			0.000*
Yes	1	9	
No	48	0	
Requirement of surgical intervention			0.000*
Yes	0	49	
No	133	0	
Total leukocyte count			0.156
<12	37	84	
>12	12	49	

## Discussion

Acute appendicitis is one of the most common causes of acute abdomen in patients admitted to the emergency room (ER) [9]. Although appendectomy has been considered the gold standard treatment for acute appendicitis, it has been paralleled with conservative management in the indoor setting [10,11]. This approach has successfully managed acute uncomplicated appendicitis. A meta-analysis by Sallinen et al. has demonstrated that conservative management is valued and even preferred by a few institutions [5]. A shared decision, however, should always be in favor. The decision to move forward with conservative management has been controversial regarding complicated appendicitis, and a consensus has yet to be reached [12,13].

Despite the conservative treatment being favored recently, there have been incidences of recurrence and treatment failures that have led few centers to incline towards surgical management. For example, research carried out at a tertiary hospital in Nagpur, India, showed a recurrence in 13% of the patients, and 25% had a treatment failure prompting surgical management [14].

Non-operative management of acute appendicitis gained even more popularity during the COVID-19 pandemic. It emerged as the first-line treatment for appendicitis in various literature from the United Kingdom (UK) [15]. Literature from Spain showed that before the pandemic, around 10% of patients presenting with appendicitis were treated with conservative management. This percentage rose to 19.2% during the COVID pandemic, clearly depicting a more conservative approach during these unforeseen circumstances [16]. Similarly, a randomized control trial carried out across 25 US centers in 2020 showed that 70% of patients had a successful outcome of treating appendicitis with antibiotics, stating that antibiotics are non-inferior to appendectomy [17].

Conservative management with antibiotics has played an essential role for patients in remote areas and other locations with the unavailability of surgical expertise. For example, Hansson et al. reported a 25-50% reduction in expenses of the antibiotic group compared to surgery [2,18]. The antibiotic treatment has also been suggested to reduce the rate of negative appendectomies, a surgical outcome usually associated with a diagnostic dilemma either due to the non-availability of a CT scan or the patient has already taken painkillers at the time of presentation and is pain-free [19].

Even with the dilemma of the unavailability of CT scans, some studies state the sensitivity and specificity of abdominal ultrasound are almost equivalent to that of CT in the hands of an experienced radiologist [11]. However, a recent meta-analysis reported the sensitivity and specificity of 69% and 81% of ultrasound abdomen, respectively. Furthermore, it showed no role in the diagnostic pathway for the diagnosis of acute appendicitis in the suspected patient. Therefore, any misdiagnosis or misclassification could be associated with an increase in failure of conservative management [20].

The same study stated that a CT scan of the abdomen was the investigation of choice with sensitivity and specificity of 91% and 90%. Therefore, a more pragmatic approach could be used with the initial investigation as the ultrasound and a conditional CT scan in an inconclusive or negative examination. Leeuwenburgh et al. have demonstrated that the combination of ultrasound and CT leads to a sensitivity and specificity of 97% and 91% for the diagnosis of appendicitis [2].

Although conservative treatment is gaining popularity, our research shows that it was effective in only 26.4% of the patients. The remaining patients had recurrences or treatment failures that required surgery. One hundred twenty-three patients with treatment failure underwent an appendectomy in the same admission, while ten patients were operated on within six months of being discharged from the hospital. Emergency surgery has been associated with morbidity, but it offers the opportunity to look inside the abdomen. Researches show that Carcinoid is found in 0.43% of appendectomies and colon cancer in 0.85% of cases [21,22]. The patients admitted for conservative treatment must be followed closely over many days following the procedure. However, our research has not proven any significant difference in the duration of hospital stay.

Lastly, there were a few limitations in our study. Firstly, only the ultrasound was used to diagnose uncomplicated acute appendicitis, which might have led to misclassification and misdiagnosis of the cases leading to failure in conservative treatment. Secondly, being a retrospective study, accurate monitoring and evaluation of patients treated with conservative management were not carried out as in prospective studies or RCTs.

## Conclusions

Our research included 182 patients, but it was only practical in 26.4% of them. Hence, there was a clear indication in favor of the operative management of uncomplicated acute appendicitis. Conservative management is no doubt gaining ground, and a lot of centers are inclined towards non-operative management; therefore, further randomized controlled trials and meta-analyses should be carried out on the matter for a more conclusive verdict.
